# Collision Course: A Decade of Traumatic Brain Injury Trends and the Impact of Urban Safety Initiatives in Eastern Massachusetts

**DOI:** 10.3390/jcm14165825

**Published:** 2025-08-18

**Authors:** Maxwell B. Baker, Himani Sood, Dhanesh D. Binda, Erin Dienes, Ala Nozari, Tejal S. Brahmbhatt, Kushak Suchdev, Ali Daneshmand

**Affiliations:** 1 Department of Anesthesiology, Boston University Chobanian & Avedisian School of Medicine, Boston, MA 02218, USA; ddb96@bu.edu (D.D.B.); erin.dienes@bmc.org (E.D.); ala.nozari@bmc.org (A.N.); 2 Department of Emergency Medicine, University of Vermont Larner College of Medicine, Burlington, VT 054052, USA; 3 Department of Neurology, Boston University Chobanian & Avedisian School of Medicine, Boston, MA 02118, USA; hsood@bu.edu (H.S.); kushaksuchdev@gmail.com (K.S.); alid@bu.edu (A.D.); 4 Department of Anesthesiology, Montefiore Einstein Medical Center, Bronx, NY 10467, USA; 5 Department of Surgery, Boston University Chobanian & Avedisian School of Medicine, Boston, MA 02118, USA; tejal.brahmbhatt@cshs.org; 6 Department of Surgery, Cedars Sinai Medical Center, Los Angeles, CA 90048, USA

**Keywords:** traumatic brain injury, motorcycle accidents, pedestrian injuries, urban health, disability evaluation, retrospective studies, health disparities, pedestrian strike, traffic accidents prevention and control

## Abstract

**Background/Objectives:** Traumatic brain injuries (TBI) account for over a third of all injury-related deaths, predominantly due to motor vehicle collisions (MVC). This study provides a comprehensive analysis of TBI trends in Eastern Massachusetts, focusing on injuries resulting from motorcycle MVCs (mMVC), non-motorcycle MVCs (nmMVC), and pedestrian-vehicle strikes (PVS). **Methods:** A retrospective analysis was conducted on TBI patients admitted between 2010 and 2020 to Boston Medical Center. TBI severity was assessed using the Glasgow Coma Scale (GCS) on admission (mild: 13–15, moderate: 9–12, severe: 3–8), and outcomes were determined by discharge disability scales. Descriptive and inferential statistics evaluated patient profiles, TBI severity, and group differences. **Results:** Among the 2901 identified TBI cases from MVCs, 14.1% were mMVCs, 55.1% nmMVCs, and 30.8% PVS. Mortality rates were 3.7% for mMVCs, 2.1% for nmMVCs, and 8.9% for PVS. In 2017, nmMVC-related TBIs decreased by 50% and PVS-related TBIs by 35% (*p* < 0.01). The PVS group tended to be older (mean age 41.0 years) and more racially diverse, with Asian patients overrepresented. The mMVC group had a significantly skewed gender distribution, with 91% male. TBI severity also varied significantly, with the mMVC and PVS groups experiencing more severe TBIs compared to the nmMVC group (*p* < 0.001). Discharge outcomes, as assessed by the Cerebral Performance Category (CPC) scale, differed across cohorts (*p* = 0.0005), with the PVS group showing the most severe outcomes and the nmMVC group demonstrating the highest rate of return to previous function (CPC 0: 5.6%). **Conclusions:** Our study revealed significant differences in injury severity and outcomes based on the type of vehicular collision. Notably, Asian patients were disproportionately affected by PVS. Older PVS patients exhibited higher mortality rates, while severe TBIs were more common among male mMVC patients. In contrast, nmMVC patients showed better recovery outcomes. Coinciding with the implementation of Boston’s Vision Zero initiative in 2017, decreases in both nmMVC-related and PVS-related TBIs were observed; however, other contributing factors may have also influenced this decline. These findings highlight the urgent need for targeted public health strategies to mitigate TBI risks across diverse populations.

## 1. Introduction

Traumatic brain injuries (TBIs) represent a significant national health burden, accounting for approximately one-third of all injury-related deaths in the United States (U.S.) [1,2,3]. Such injuries not only result in devastating outcomes for patients but also place considerable strain on their families and the healthcare system [4]. While a diverse array of mechanisms can lead to TBIs, motor vehicle collisions (MVCs) and pedestrian-vehicle strikes (PVS) have garnered particular attention as TBIs are a leading cause of mortality and severe disability, especially in young men [5]. Emerging research highlights distinct patterns in TBIs resulting from motorcycle MVCs (mMVC), non-motorcycle MVCs (nmMVC), and PVS [6,7,8,9]. Given the high energy transfer associated with these injuries, understanding these variations is critical for optimizing triage and crafting targeted treatment plans.

In the U.S., it is estimated that 91% of commuters use personal vehicles such as cars and motorcycles, each presenting unique risk factors due to differences in mass, acceleration, maneuverability, and occupant protection [10]. Modern cars, SUVs, and trucks enhance occupant safety with features like crumple zones and airbags but sacrifice some maneuverability due to their size. In contrast, motorcycles offer greater agility and acceleration but lack protective structures, exposing riders to higher impact forces. Pedestrians are at the highest risk of severe injury and death in vehicle collisions, as they lack any physical protection, whereas motorcyclists may wear helmets and protective gear [11]. Furthermore, demographic factors such as age, gender, race, and socioeconomic status significantly influence TBI outcomes and recovery paths [12].

In December 2015, the City of Boston introduced its Vision Zero initiative, which aims to eliminate traffic fatalities and severe injuries by 2030 through safer street design, enforcement, and community engagement [13]. Key measures include reducing the city’s default speed limit from 30 to 25 mph (implemented on 9 January 2017), enacting the Neighborhood Slow Streets program to calm traffic in residential areas, and expanding protected bike lanes and safer pedestrian crossings.

With over 131,000 emergency department visits and 2000 trauma activations annually, our American College of Surgeons (ACS)-verified Level 1 trauma center ranks 12th in patient volume among all Level 1 trauma centers in the U.S., providing a valuable source of data through its high patient volume in emergency, trauma, and perioperative care. Our study sought to compare the characteristics of TBI patients who presented to our institution between 2010 and 2020 across the three distinct mechanisms of mMVC, nmMVC, and PVS. Additionally, we aimed to evaluate the impact of Boston’s Vision Zero initiative on the incidence of TBI, while independently analyzing the distribution and severity of TBIs across different mechanisms of injury.

## 2. Materials and Methods

### 2.1. Study Design

We conducted a retrospective chart review to investigate demographic differences and functional outcomes of TBIs among patients involved in mMVCs, nmMVCs, and PVS who were admitted to our ACS-verified Level 1 trauma center from 1 January 2010 to 31 December 2020. Our research study was reviewed and considered exempt by the Boston Medical Center and Boston University Medical Campus Institutional Review Board.

### 2.2. Data Collection

Patient data were manually extracted from institutional electronic medical records (EMR) and recorded in a local TBI registry. The study categorized patients into three cohorts: mMVC, involving motorcycle collisions; nmMVC, involving car collisions; and PVS, involving pedestrians (excluding bicyclists) struck by vehicles.

### 2.3. TBI Severity Determination

The severity of each patient’s TBI was determined using the initial Glasgow Coma Scale (GCS) score upon hospital admission. Severity was categorized by standard convention as mild for a GCS score of 13–15, moderate for a GCS score of 9–12, and severe for a GCS score of 3–8.

### 2.4. Discharge Disability Evaluation

At discharge, patients’ functional outcomes were evaluated using the Cerebral Performance Category (CPC) disability scale that classified their condition into no disability or discharge with previous level of function (CPC 0), temporary disability with expected to return to previous level of function (CPC 1), moderate disability with expected ability for self-care (CPC 2), severe disability (CPC 3), persistent vegetative state (CPC 4), or brain death or death (CPC 5) [14]. CPC scores were assigned retrospectively by the authors based on interpretation of clinical documentation and discharge summaries in the EMR.

### 2.5. Data Analysis

Descriptive statistics were utilized to characterize the patient profiles within the mMVC, nmMVC, and PVS cohorts. The incidence of TBI severity was calculated for each group based on categorized GCS scores. The outcome for each cohort was determined based on the discharge CPC disability scale. Inferential statistics, including chi-square tests of independence and Fisher’s exact test (when bin sizes were small), were used to assess distributional differences in gender, race, ethnicity, and TBI severity between the cohorts. Analysis of Variance (ANOVA) was used to determine whether ages differed significantly between mechanisms of injury. Tukey Family Confidence intervals were employed to control the familywise error rate when comparing multiple groups. A multinomial model was used to determine if race was a risk factor for suffering a TBI by PVS or mMVC as compared to nmMVC. To examine potential changes in TBI incidence over time, a change point analysis was conducted on each cohort. All statistical analyses were conducted using the R version 4.4.0 software package, with a significance level set at *p* < 0.05 [15].

## 3. Results

**Figure 1:**
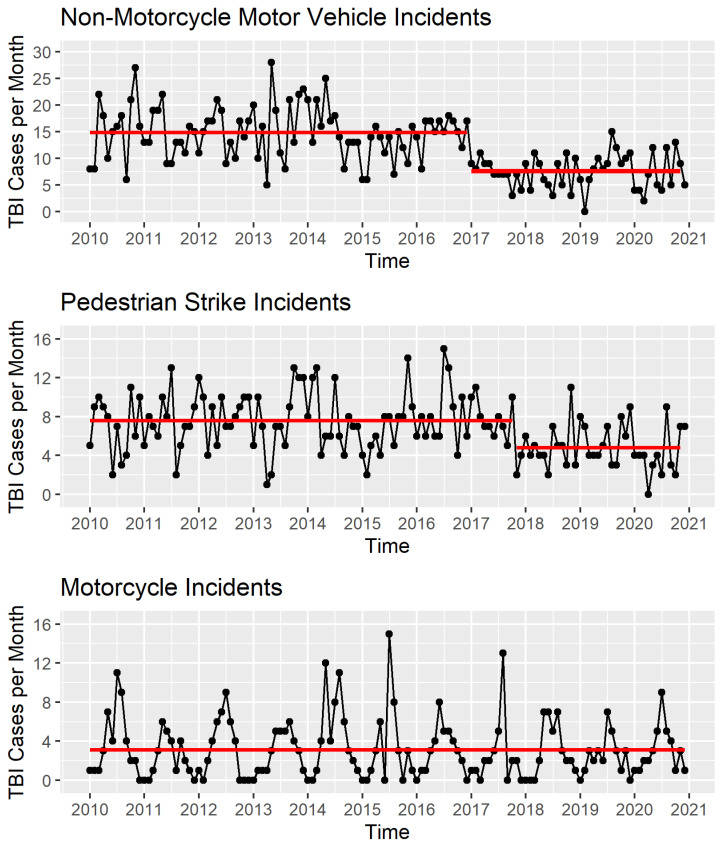
TBI incidence for the nmMVC, PVS, and mMVC cohorts from 2010 to 2020 at Boston Medical Center. A significant reduction in the number of nmMVC and PVS TBI admissions in 2017 coincides with the implementation of speed restrictions under Boston’s Vision Zero initiative. TBI, traumatic brain injury; mMVC, motorcycle motor vehicle collisions; nmMVC, non-motorcycle motor vehicle collisions; PVS, pedestrian-vehicle strikes. The red lines in the figures represent averages.

Our analysis encompassed a total of 2901 MVC-related TBI patients between 2010 and 2020, with 408 patients in the mMVC group, 1599 patients in the nmMVC group, and 894 in the PVS group. There was a marked reduction in TBI incidence within both the nmMVC and PVS cohorts beginning in 2017 (January and October, respectively) (Figure 1). Following these changepoints, TBIs from nmMVC collisions decreased by approximately 50%, and PVS-related TBIs decreased by around 35% (*p* < 0.01). In contrast, no significant change was observed in the incidence of motorcycle collisions over time.

### 3.1. Age and Gender

**Figure 2:**
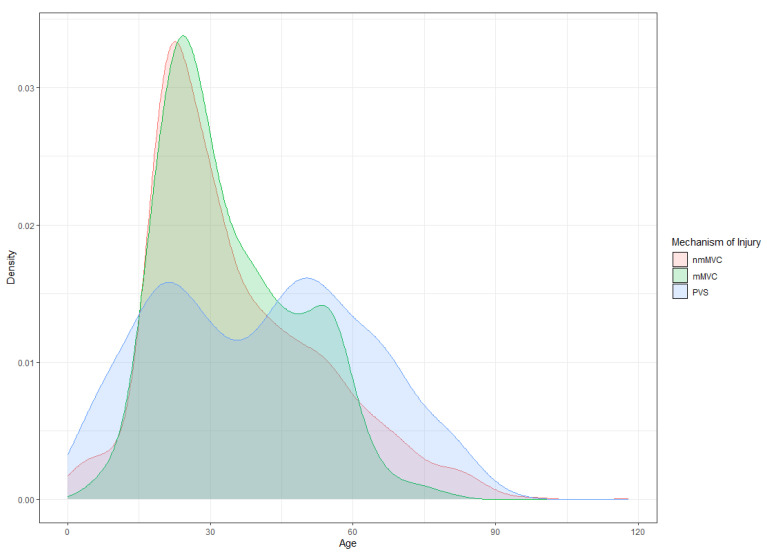
Density plots of age by mechanism of injury. The age distribution in the PVS group was bimodal, peaking in the mid-20 s and early 50 s, while the mMVC group had a less pronounced peak around ages 55–60. mMVC, motorcycle motor vehicle collisions; nmMVC, non-motorcycle motor vehicle collisions; PVS, pedestrian-vehicle strikes.

The mean age of patients was 34.3 years in the mMVC cohort, 35.2 years in the nmMVC cohort, and 41.0 years in the PVS cohort. Tukey Family Confidence intervals indicated no significant age difference between the mMVC and nmMVC groups, but the PVS group was significantly older, averaging at least 4 years more than the other two groups. The age distribution in the PVS group was notably bimodal, with peaks in the mid-20 s and early 50 s (Figure 2). Similarly, the mMVC group showed a secondary peak around ages 55–60, though it was less pronounced compared to the PVS group.

Gender distribution varied significantly across cohorts. In the mMVC group, 91.0% were male compared to 9.0% female. The nmMVC and PVS groups demonstrated more balanced distributions, with 62.0% male and 38.0% female, and 61.0% male and 39.0% female, respectively. A chi-square test confirmed significant differences in gender distribution across all groups (*p* < 0.001). Additionally, a z-score test showed significant differences between the mMVC and nmMVC groups (*p* < 0.001).

### 3.2. Race/Ethnicity and Location

The racial and ethnic composition of the cohorts revealed notable group differences (Figure S1). Patients in the mMVC group were predominantly White or Black, with 37.7% of patients identifying as White, 37.3% as Black, 0.7% as Asian, 21.6% as Other, while the race/ethnicity of 2.7% was unknown. The nmMVC group displayed a comparable racial diversity, consisting of 40.5% White, 35.8% Black, 3.1% Asian, 18.4% Other, and 2.2% unknown. The PVS group showed varied diversity: 38.5% Black, 33.3% White, 8.6% Asian, 18.3% other, and 1.2% unknown. A chi-squared test confirmed that these racial distributions were statistically significant (*p* < 0.05). A multinomial analysis examining the risk of PVS by race showed that Asian patients were 2.4 times more likely to be involved in a PVS compared to a nmMVC (Table 1).

Examining the injuries by county, Suffolk exhibited a notably higher percentage of PVS patients at 39.0%, compared to 30.8% across the entire database, and a lower percentage of nmMVC patients at 46.4%, versus 55.1% overall (Figure S2). In Suffolk, Middlesex, and Norfolk, the three counties with the highest proportions of Asian residents according to census data, Asian patients were most frequently admitted as victims of PVS (Figure S3).

Ethnicity analysis showed that 17.2% of the mMVC group identified as Hispanic/Latino, compared to 14.6% in the nmMVC group, and 16.2% in the PVS group. Z-score testing revealed no significant differences in ethnicity between the mMVC and nmMVC groups (*p* = 0.23), and chi-squared testing found no significant differences across all three groups (*p* = 0.157). These findings suggest that self-reported ethnicity did not significantly differentiate the mMVC, nmMVC, and PVS groups in this study.

### 3.3. TBI Severity Incidence

The severity of TBIs, assessed by admission GCS scores, varied among the groups. In the mMVC group, 82.8% experienced mild TBIs, 2.2% moderate, and 13.0% severe (Figure S4). The nmMVC group had 89.3% mild TBIs, 2.6% moderate, and 7.4% severe. The PVS group showed 80.9% mild TBIs, 2.6% moderate, and 14.8% severe. The incidence of mild and severe TBIs was similar in the mMVC and PVS groups, while the nmMVC group mainly had mild TBIs, with fewer cases of severe TBI. These findings, confirmed by a chi-square test (*p* < 0.001), indicate significant differences in TBI severity across the cohorts, even after adjusting for multiple comparisons.

Asian and White patients presented with significantly lower average GCS scores on admission compared to Black patients. Among those admitted with a GCS score indicative of “mild” TBIs, Asian patients had a mortality rate of 4%, which was 10 times higher than that of any other racial group. In the subset of patients with a “moderate” GCS score on admission, the mortality rate among Asian patients was 40% (2 out of 5), five times higher than that of White patients. However, among patients admitted with severe TBI, mortality rates did not differ significantly across racial groups.

### 3.4. Discharge Disability Outcomes

**Figure 3:**
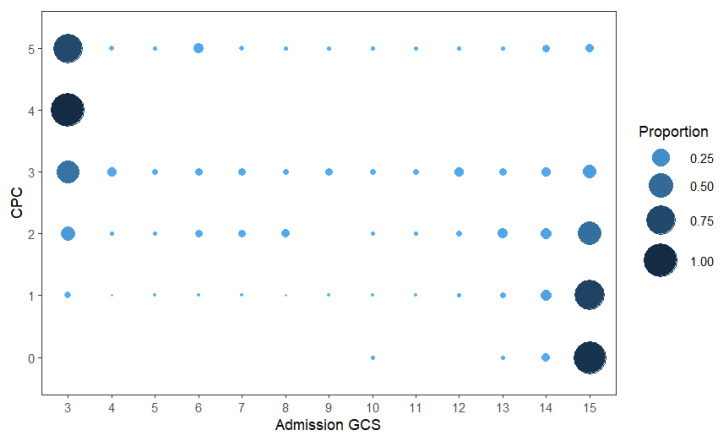
Analysis of discharge disability outcomes using the CPC scale compared to admission GCS scores. Patients with lower GCS scores tended to experience worse outcomes, whereas those with higher GCS scores typically had more favorable outcomes. CPC, Cerebral Performance Category; GCS, Glasgow Coma Scale.

The analysis of discharge disability outcomes highlights distinct patterns across the groups (Figure 3).

Table S1 summarizes the discharge disability among patients with TBIs from mMVCs, nmMVCs, and PVS. In the mMVC group, the majority of patients experienced temporary disability (CPC 1, 81.6%), followed by moderate disability (CPC 2, 4.2%). A smaller percentage of patients were noted to have severe disability (CPC 3, 2.5%), be in a persistent vegetative state (CPC 4, 0.2%), or be deceased (CPC 5, 3.7%). Only 2.0% of patients returned to their previous function (CPC 0). The neurological outcome of 5.9% of these patients remains unknown.

In the nmMVC group, temporary disability (CPC 1, 83.6%) was the most common outcome. A notably higher proportion of patients returned to their previous level of function (CPC 0, 5.6%) compared to the mMVC and PVS groups. Other outcomes included 2.5% CPC 2, 1.8% CPC 3, 0.0% CPC 4, and 2.1% CPC 5, with no patients in a persistent vegetative state. The outcome for 4.5% of nmMVC patients is unknown. While the PVS group showed a significant proportion of patients experiencing temporary disability (CPC 1, 77.3%), these patients also had a higher mortality rate (CPC 5, 8.9%) compared to the mMVC and nmMVC groups. Other outcomes included 3.1% CPC 0, 4.3% CPC 2, 1.5% CPC 3, and 0.0% CPC 4, with 4.9% of cases remaining unknown.

Fisher’s exact test for discharge disability outcomes across the mMVC, nmMVC, and PVS cohorts demonstrated statistical significance (*p* = 0.0005), indicating varied patterns in functional outcomes and mortality. Notably, the mortality rate in the PVS group was over twice that of any other group, highlighting differences in the severity of injuries among the cohorts. The nmMVC group had the highest proportions of CPC 0 and CPC 1 levels of function at discharge.

## 4. Discussion

Our retrospective study of 2901 MVC-related TBIs at a high-volume urban trauma center in Eastern Massachusetts revealed significant demographic, severity, and outcome differences based on collision type. Asian patients were overrepresented in PVS but less involved in other MVC types. PVS patients were older, with higher mortality and more severe injuries, while male mMVC patients often sustained severe TBIs. In contrast, the nmMVC group, with a more balanced gender distribution, had better recovery outcomes and mostly mild TBIs. A significant decrease in the number of TBIs resulting from nmMVC and PVS in 2017 coincided with the implementation of speed restrictions under Boston’s Vision Zero initiative. However, it is unclear if this was the sole factor, as other variables may have also played a role in reducing the severity and frequency of TBIs. These findings underscore the need for tailored medical and rehabilitation strategies for different demographic groups and collision types.

### 4.1. Demographic Findings

The racial distribution varied significantly among the groups in our study. In 2023, Caucasians made up the majority of motorcycle riders (88%), followed by African Americans (7%), Hispanics (4%), and Asian and Pacific Islanders (1%) [16]. In our study, Asian patients showed the greatest variation across the three mechanisms of injury, comprising only 0.7% of mMVC patients, 3.0% of nmMVC patients, and 8.6% of PVS patients. Asian patients were 2.4 times more likely to be involved in a PVS than in an nmMVC, suggesting cultural, socioeconomic, behavioral, or environmental factors may influence the lower incidence of motorcycle-related TBIs and the higher proportion of PVS incidents in this cohort. For instance, the MASALA Study found that for South Asian American men, an increase in walkability was associated with increased walking for transport [17]. Another study in Hong Kong found that walking for transport among elderly residents was attributed to several factors, including the city’s high-density, compact urban layout, abundant destinations within easy reach, the availability of an extensive and efficient public transport system, relatively low car ownership rates, and cultural influences [18]. Our data showed that Suffolk County had a notably higher percentage of PVS patients and a lower percentage of nmMVC patients, possibly due to its higher walkability and population density in the city of Boston [19].

While all groups were predominantly male, those in the mMVC group were significantly more likely to be male compared to the nmMVC and PVS groups. These results are consistent with prior research on traumatic injury prevention, emphasizing the heightened risk among male motorcycle operators [20,21]. The analysis of age also showed a statistically significant difference, with the average age in the PVS group being at least 4 years older than that in both the mMVC and nmMVC groups. However, the PVS group displayed a bimodal age distribution, suggesting that both younger and older adults are more inclined to walk rather than drive or use public transportation, possibly due to behavioral and socioeconomic factors [22,23,24,25]. Multinomial analysis revealed that each additional year of age slightly reduced the likelihood of being in an mMVC versus an nmMVC by 0.2%, though not significantly, while each additional year increased the odds of being in a PVS by 1.7%.

The observed increase in PVS incidence with age may be attributed to multiple age-related factors. Older adults are more likely to rely on walking for transportation due to medical, financial, or lifestyle constraints, particularly in dense urban environments [26]. Additionally, age-related declines in visual acuity, hearing, mobility, and reaction time may impair their ability to safely navigate traffic environments, increasing their risk of being struck [27]. These factors, combined with a higher likelihood of sustaining severe injuries upon impact, help explain both the elevated incidence and mortality observed in older pedestrians. A 2021 study by Islam and associates found that motorcyclists’ age significantly influences their safety perception and risk assessment capabilities, thereby affecting their involvement in risky behaviors [28]. Furthermore, another study highlighted that motorcycle fatality rates are rising, particularly among riders aged 60 and older, with those aged 18 to 29 experiencing the highest overall fatality rates [29]. This trend could explain why the mMVC group showed a secondary, smaller peak for the mechanism of injury around ages 55–60.

### 4.2. TBI Severity and Admission GCS Score

The severity of TBIs displayed distinct patterns across the groups. Patients in the mMVC and PVS categories exhibited a significantly higher percentage of severe TBIs compared to those in nmMVCs. Previous research has validated such observations as pedestrians have a mortality rate nearly twice that of motor vehicle occupants, often presenting with more severe injuries and a higher rate of severe head injuries [30]. The difference likely reflects the higher energy transfer in motorcycle and pedestrian incidents, where protective barriers like vehicle crumple zones and restraint systems are absent [19]. Without these safeguards, pedestrians, bicyclists, and motorcyclists become especially vulnerable to severe intra- and extracranial injuries such as complex facial or orthopedic fractures, which may require timely repair [30,31]. Additional studies have linked the severity of TBI to specific collision characteristics, such as lateral impacts and collisions with fixed objects, highlighting the critical role of safety restraints like seatbelts in mitigating brain injury risks [32]. The initial GCS score has also proven to be a reliable indicator of mortality risk in these scenarios, underscoring its value in emergency settings for assessment and prognosis planning [33].

### 4.3. Discharge Outcomes

Discharge outcomes varied significantly between the groups, with the highest mortality rates observed among PVS patients, likely due to the severity of injuries and the older age distribution within this group [34]. Patients with lower GCS scores (3–8) were more likely to have poor outcomes (CPC 4–5), while those with higher GCS scores (13–15) generally had better outcomes (CPC 0–2). Although some poor outcomes were still observed among patients with higher GCS scores, the overall trend showed that higher GCS scores at admission were associated with improved discharge outcomes. It is important to note the limitations of using discharge outcomes for long-term prognostic decisions in moderate to severe TBI patients, as many can show substantial recovery months after discharge. For instance, by 12 months post-injury, 52.4% of severe TBI and 75% of moderate TBI patients achieved favorable outcomes, compared to just 12.4% and 41% at 2 weeks, highlighting the need for continued monitoring and reassessment [35]. Due to the small sample sizes, we were unable to draw meaningful conclusions about mortality rates in Asian patients with mild or moderate TBI. Although the data suggest higher mortality rates in this group compared to others, the limited number of cases prevents us from making definitive statements. However, clear racial and ethnic differences do exist in TBI-related hospitalizations and deaths. For instance, TBI-related deaths disproportionately affect Black populations [36]. Research indicates that differences in healthcare utilization among adults with TBIs are closely linked to insurance coverage, race, and ethnicity, suggesting that access to and use of healthcare services significantly influence TBI prognosis and recovery [37].

While our findings suggest that PVS patients had more severe TBIs and worse discharge outcomes compared to other mechanisms, these results must be interpreted in light of potential confounding factors. The PVS cohort was, on average, older and may have had a greater burden of pre-existing medical conditions that independently increase the risk of mortality and poorer recovery following TBI. Moreover, differences in healthcare access or response times may have also influenced outcomes. Our current dataset did not include systematic information on comorbidities, functional baseline, or prehospital care, which limits our ability to fully disentangle the effects of injury mechanism from patient characteristics. Future studies should incorporate risk-adjusted models that account for age, comorbidities, and social determinants of health to provide a more nuanced understanding of TBI outcomes across different collision types.

### 4.4. Implications for Prevention and Treatment

The observed demographic differences among TBI patients in Eastern Massachusetts underscore the need for tailored injury prevention strategies. Public health campaigns, for instance, could target the most affected demographic groups, such as through motorcycle safety initiatives, to enhance effectiveness [38]. Interestingly, there was a significant shift in nmMVC- and PVS-related TBIs between December 2016 and January 2017. While investigating this unique reduction amid broader national trends, a targeted city-wide intervention like Boston’s Vision Zero initiative emerged as a likely factor in reducing nmMVCs. Although no study has directly analyzed injury characteristics in relation to the Vision Zero program, research on its overall impact aligns with our findings on the trends of TBIs linked to vehicle collisions in the city [13]. For example, one study found that Vision Zero led to a reduction in mean vehicle speed across the city and significantly lowered the odds of vehicles exceeding 25, 30, and 35 mph by 2.9%, 8.5%, and 29.3%, respectively [39]. Public awareness campaigns and the promotion of alternative transportation options may have further contributed to a reduction in crashes citywide.

Additionally, the variation in TBI severity and outcomes underscores the importance of specialized trauma care and rehabilitation services, tailored to meet the unique needs of different patient groups [40]. Research emphasizes the necessity for integrated approaches to enhance prevention, clinical care, and TBI studies, including understanding the varied causes of TBI across different settings and developing region-specific prevention strategies [41]. For example, in low and middle-income countries, road traffic collisions involving vulnerable users like motorcyclists and pedestrians predominate, while in high-income countries, falls, especially among the elderly, are more common [42]. This insight suggests that prevention strategies should be specifically tailored to the prevalent causes in each region. Research also discusses the challenges in TBI prevention, clinical care, and research, highlighting the importance of targeted prevention measures based on risk factors such as frailty and alcohol misuse. It notes that older TBI patients, often injured in low-energy falls, are less likely to receive critical care or emergency interventions compared to those involved in high-energy incidents such as road traffic collisions, emphasizing the need for equitable and appropriate clinical care across all age groups and injury mechanisms [43].

While our analysis identified that Asian patients were disproportionately represented among PVS cases, the absence of granular data on neighborhood walkability, transportation reliance, language proficiency, or access to safe pedestrian infrastructure limited our ability to examine the underlying causes of this disparity. These structural and environmental factors may significantly shape pedestrian risk and health outcomes in urban settings. Further research should incorporate geospatial and sociodemographic datasets to better elucidate the mechanisms driving these disparities and guide targeted, equity-focused public health interventions.

### 4.5. Limitations and Future Research

Our study has several limitations that warrant further investigation. The retrospective design restricts our ability to establish causality between injury mechanisms and TBI outcomes, particularly in evaluating public health interventions such as Boston’s Vision Zero initiative. While we observed a temporal association between the program’s implementation and a decline in nmMVC- and PVS-related TBIs beginning in 2017, other concurrent factors, such as statewide traffic policy changes, changes in emergency medical services protocols, infrastructure improvements, or regional socioeconomic shifts, may have contributed to this trend. Studies employing time-series or interrupted time-series analyses are needed to rigorously evaluate the independent impact of Vision Zero policies on TBI incidence.

Additionally, our single-center dataset, drawn from a high-volume urban trauma center, may not be representative of TBI demographics across Eastern Massachusetts, particularly in suburban or rural areas, thereby limiting the generalizability of our findings. However, our institution regularly receives trauma patients transferred from outside hospitals across New England and also serves as a regional destination for patients flown directly from the scene of injury by helicopter. These interfacility transfers and direct field transports reflect the institution’s role as a major referral center for severe trauma across the region and contribute to a diverse patient population. Even though this enhances the clinical breadth of our sample, the absence of a formal control group or comparison dataset from other institutions limits our ability to assess whether these trends are generalizable across broader populations. Moreover, while we used the CPC scale to assess discharge outcomes, long-term functional recovery would provide a more accurate assessment of TBI prognosis, as initial injury severity can be difficult to fully gauge, and secondary brain injury may occur weeks later [44]. Discharge CPC scores capture only immediate post-injury disability and may not reflect long-term recovery. Future studies with longitudinal follow-up and validated outcome measures (e.g., GOSE or FIM) are needed to better characterize recovery patterns and guide personalized rehabilitation strategies.

Furthermore, multi-center, prospective designs and population-level data research are needed to account for potential confounders, assess long-term outcomes, and explore the role of socioeconomic, cultural, and behavioral factors. Evaluating the efficacy of tailored preventive measures and clinical interventions for high-risk groups remains essential, and multidisciplinary collaboration may enhance innovation in TBI management and prevention.

## 5. Conclusions

Our ten-year retrospective study of TBI victims at a high-volume Level 1 trauma center highlights key differences in demographics, injury severity, and outcomes across collision types. Asian patients had a higher likelihood of involvement in PVS, potentially due to cultural or socioeconomic factors. Mortality was higher in older PVS patients, severe TBIs were more common in male patients from mMVC, and better recovery outcomes were seen in the more gender-balanced nmMVC group. These findings underscore the need for tailored medical, rehabilitation, and public health strategies, such as Boston’s Vision Zero, to address specific risks and reduce TBI severity.

## Figures and Tables

**Table 1:** A multinomial model using White ethnicity as the reference category and non-motorcycle collisions (nmMVC) as the reference outcome. mMVC, motorcycle motor vehicle collisions; PVS, pedestrian-vehicle strikes.

Mechanism of Injury	Term	Estimate	Standard Error	Statistic	*p*-Value	95% Lower Bound	95% Upper Bound
mMVC	(Intercept)	0.257	0.607	−2.229	0.026	0.079	0.849
mMVC	Age	0.998	0.003	−0.675	0.500	0.992	1.004
mMVC	Asian	0.257	0.602	−2.258	0.024	0.079	0.836
mMVC	Black/ African American	1.102	0.607	0.157	0.875	0.335	3.618
mMVC	Other	1.236	0.688	0.305	0.760	0.320	4.752
mMVC	Unknown	1.314	0.601	0.452	0.651	0.404	4.268
PVS	(Intercept)	0.221	0.209	−7.213	0.000	0.147	0.333
PVS	Age	1.017	0.002	7.480	0.000	1.013	1.022
PVS	Asian	3.415	0.199	6.172	0.000	2.312	5.043
PVS	Black/ African American	1.506	0.211	1.941	0.052	0.996	2.277
PVS	Other	1.398	0.395	0.847	0.397	0.644	3.031
PVS	Unknown	0.728	0.199	−1.595	0.111	0.493	1.075

## Data Availability

Data access is available upon written request.

## Supplementary Materials

The following supporting information can be downloaded at https://www.mdpi.com/article/10.3390/jcm14165825/s1, Figure S1: Stacked bar chart showing injury mechanisms by race, with numbers inside each bar representing patient counts. PVS was the leading cause of TBI in Asian patients, who were 2.4 times more likely to be struck by a vehicle than to be involved in a nmMVC. TBI, traumatic brain injury; mMVC, motorcycle motor vehicle collisions; nmMVC, non-motorcycle motor vehicle collisions; PVS, pedestrian-vehicle strikes. Figure S2: Stacked bar chart of the proportion of mechanisms of injury within each county. Suffolk County had a higher proportion of PVS patients and a lower proportion of nmMVC patients compared to the overall database. mMVC, motorcycle motor vehicle collisions; nmMVC, non-motorcycle motor vehicle collisions; PVS, pedestrian-vehicle strikes. Figure S3: Stacked bar chart shows the proportion of injury mechanisms within each race by county. Asian patients were most often admitted due to PVS incidents in Suffolk, Middlesex, and Norfolk. mMVC, motorcycle motor vehicle collisions; nmMVC, non-motorcycle motor vehicle collisions; PVS, pedestrian-vehicle strikes. Figure S4: Stacked bar chart of the percent of severity of TBI as determined by GCS on admission within each mechanism of injury. The mMVC and PVS groups had similar rates of mild and severe TBIs, whereas the nmMVC group predominantly experienced mild TBIs. TBI, traumatic brain injury; GCS, Glasgow Coma Scale; mMVC, motorcycle motor vehicle collisions; nmMVC, non-motorcycle motor vehicle collisions; PVS, pedestrian-vehicle strikes. Table S1: Discharge disability among patients with TBIs from mMVCs, nmMVCs, and PVS represented as count (%). mMVC, motorcycle motor vehicle collisions; nmMVC, non-motorcycle motor vehicle collisions; PVS, pedestrian-vehicle strikes; CPC, Cerebral Performance Category.
