# Supplementary material for: Collision Course: A Decade of Traumatic Brain Injury Trends and the Impact of Urban Safety Initiatives in Eastern Massachusetts
Source: J Clin Med. 2025 Aug 18;14(16):5825. doi: 10.3390/jcm14165825 (PMC12386731; doi:10.3390/jcm14165825)
Supplement: Supplementary file 1 [file jcm-14-05825-s001.zip › jcm-3746658-supplementary.pdf]

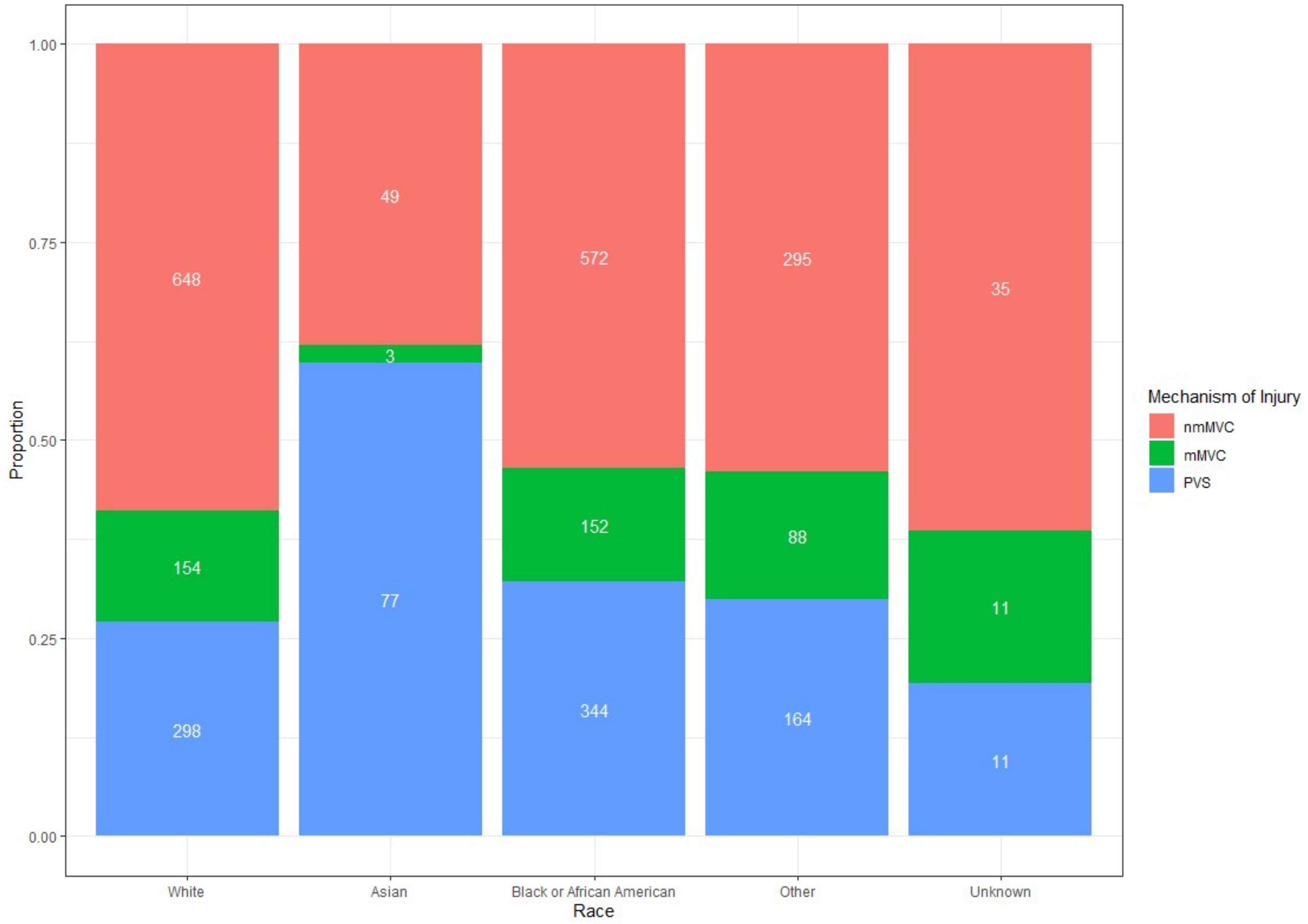

Figure S1. Stacked bar chart showing injury mechanisms by race, with numbers inside each bar representing patient counts. PVS was the leading cause of TBI in Asian patients, who were 2.4 times more likely to be struck by a vehicle than to be involved in a nmMVC. TBI, traumatic brain injury; mMVC, motorcycle motor vehicle collisions; nmMVC, non-motorcycle motor vehicle collisions; PVS, pedestrian-vehicle strikes.

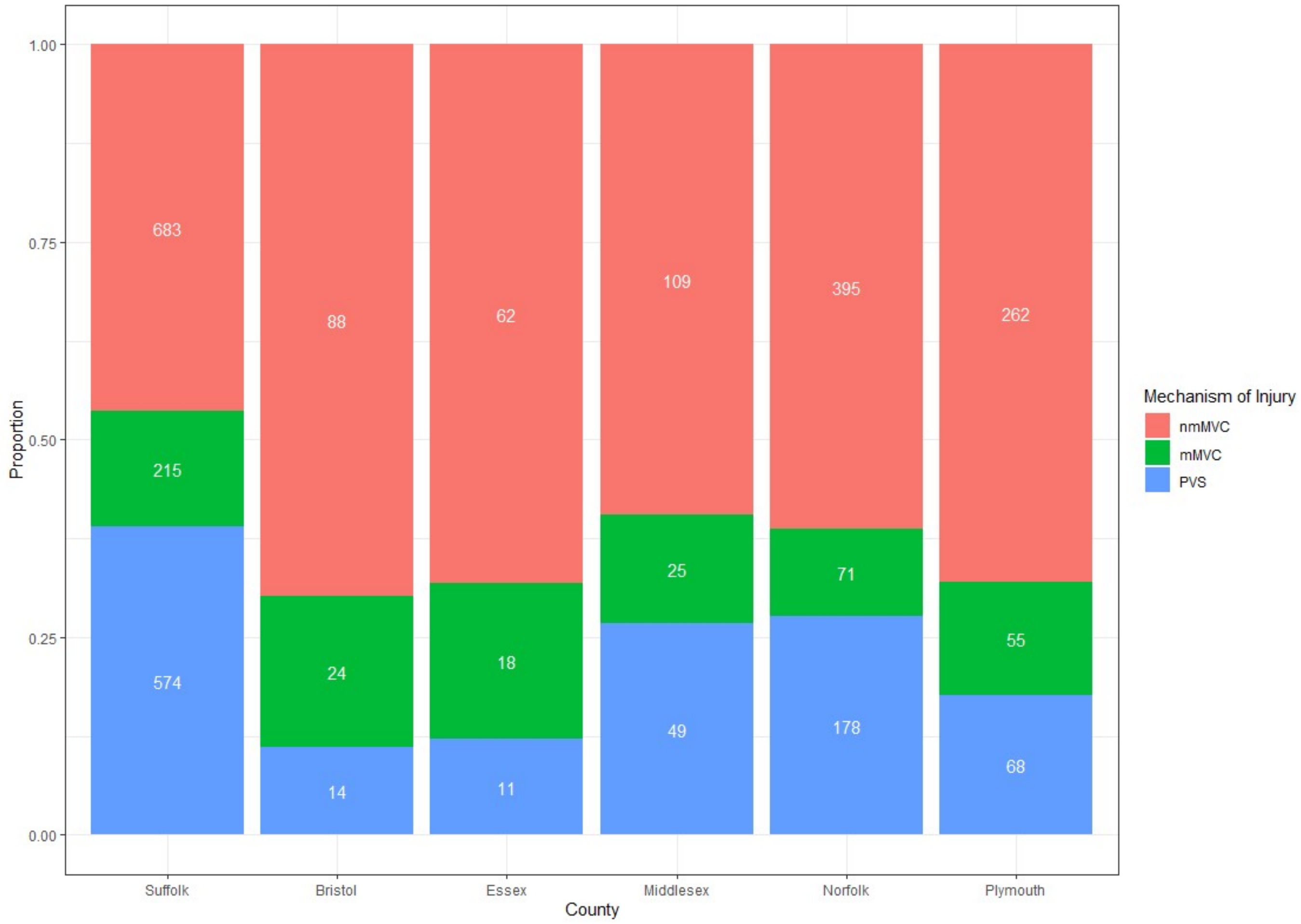

Figure S2. Stacked bar chart of the proportion of mechanisms of injury within each county. Suffolk County had a higher proportion of PVS patients and a lower proportion of nmMVC patients compared to the overall database. mMVC, motorcycle motor vehicle collisions; nmMVC, non-motorcycle motor vehicle collisions; PVS, pedestrian-vehicle strikes.

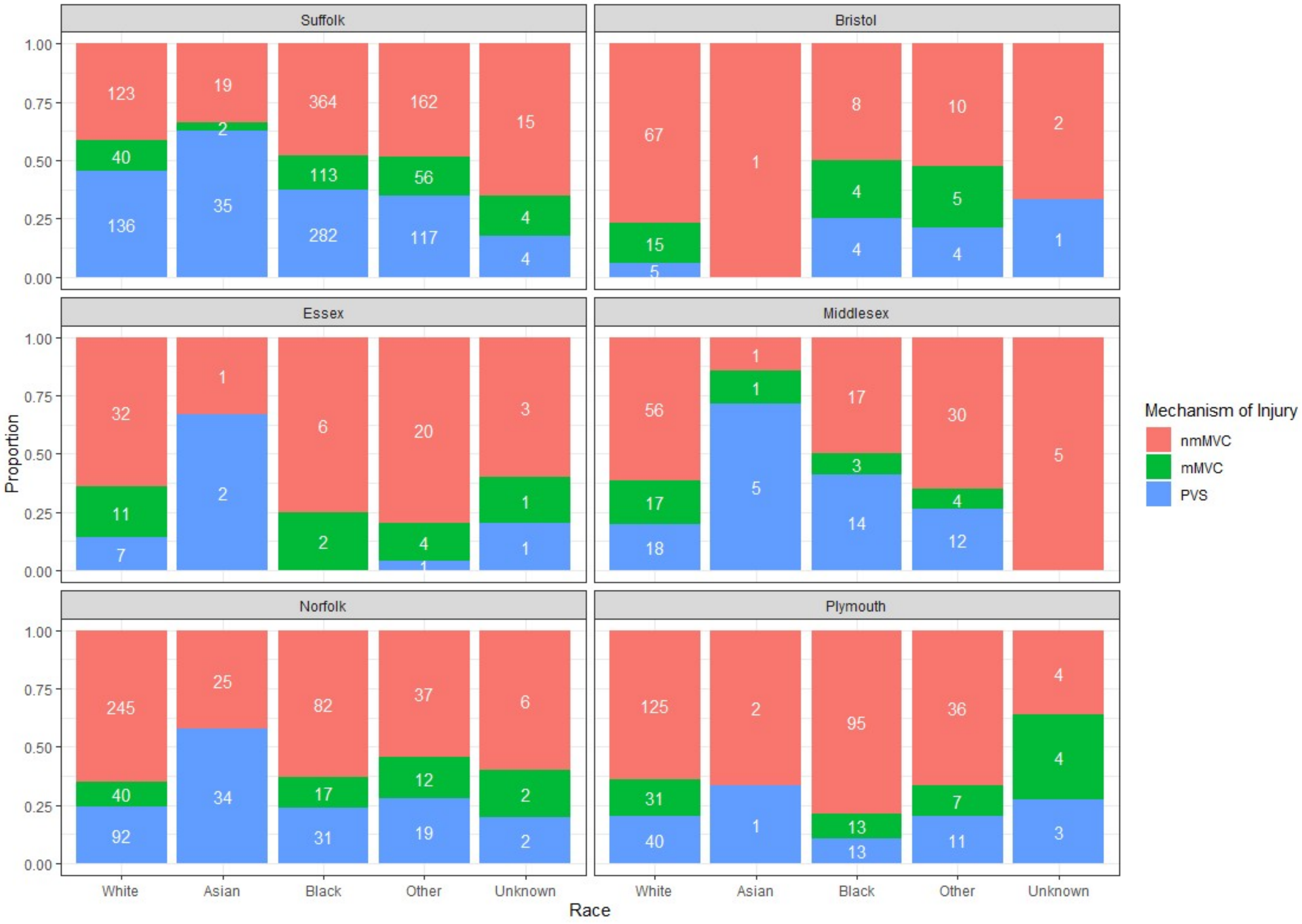

Figure S3. Stacked bar chart shows the proportion of injury mechanisms within each race by county. Asian patients were most often admitted due to PVS incidents in Suffolk, Middlesex, and Norfolk. mMVC, motorcycle motor vehicle collisions; nmMVC, non-motorcycle motor vehicle collisions; PVS, pedestrian-vehicle strikes.

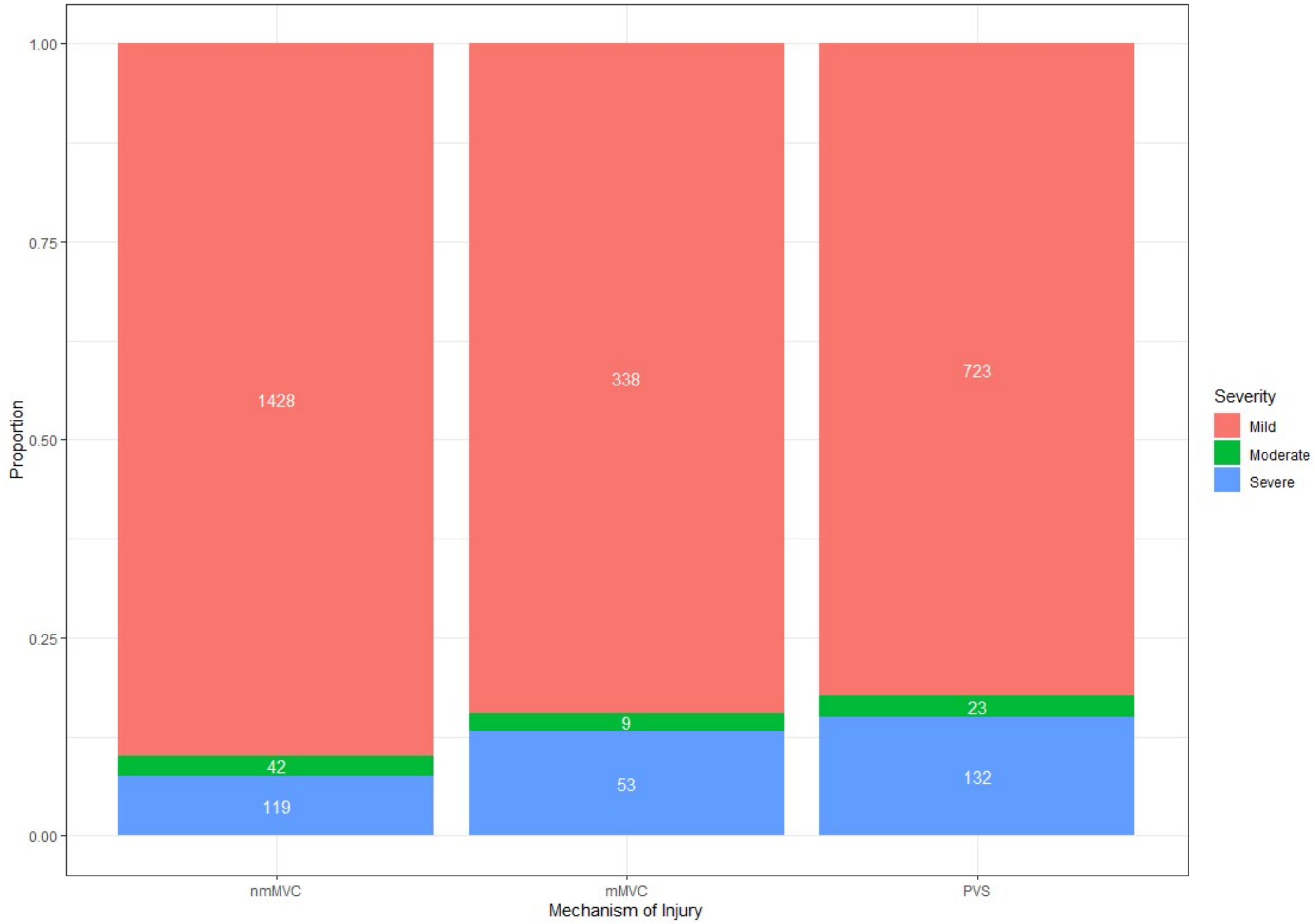

Figure S4. Stacked bar chart of the percent of severity of TBI as determined by GCS on admission within each mechanism of injury. The mMVC and PVS groups had similar rates of mild and severe TBIs, whereas the nmMVC group predominantly experienced mild TBIs. TBI, traumatic brain injury; GCS, Glasgow Coma Scale; mMVC, motorcycle motor vehicle collisions; nmMVC, non-motorcycle motor vehicle collisions; PVS, pedestrian-vehicle strikes.

Table S1. Discharge disability among patients with TBIs from mMVCs, nmMVCs, and PVS represented as count (%). mMVC, motorcycle motor vehicle collisions; nmMVC, non-motorcycle motor vehicle collisions; PVS, pedestrian-vehicle strikes; CPC, Cerebral Performance Category.

**Discharge Disability**	**mMVC (n=408)**	**nmMVC (n=1599)**	**PVS (n=894)**
Previous Level of Function (CPC 0)	8 (2.0)	89 (5.6)	28 (3.1)
Temporary Disability (CPC 1)	333 (81.6)	1337 (83.6)	691 (77.3)
Moderate Disability (CPC 2)	17 (4.2)	40 (2.5)	38 (4.3)
Severe Disability (CPC 3)	10 (2.5)	28 (1.8)	13 (1.5)
Persistent Vegetative State (CPC 4)	1 (0.2)	0 (0.0)	0 (0.0)
Dead (CPC 5)	15 (3.7)	33 (2.1)	80 (8.9)
Unknown	24 (5.9)	72 (4.5)	44 (4.9)
